# Pathological sub-analysis of a multicenter randomized controlled trial of tonsillectomy combined with steroid pulse therapy versus steroid pulse monotherapy in patients with immunoglobulin A nephropathy

**DOI:** 10.1007/s10157-015-1159-2

**Published:** 2015-09-09

**Authors:** Ritsuko Katafuchi, Tetsuya Kawamura, Kensuke Joh, Akinori Hashiguchi, Satoshi Hisano, Akira Shimizu, Yoichi Miyazaki, Masaharu Nagata, Seiichi Matsuo, The IgA nephropathy Study Group in Japan

**Affiliations:** Kidney Unit, National Fukuoka-Higashi Medical Center, 1-1-1, Chidori, Koga-City, Fukuoka 811-3195 Japan; Division of Kidney and Hypertension, Department of Internal Medicine, Jikei University School of Medicine, Tokyo, Japan; Department of Pathology, Tohoku University Graduate School of Medicine, Sendai, Japan; Department of Pathology, Keio University School of Medicine, Tokyo, Japan; Department of Pathology, Faculty of Medicine, Fukuoka University, Fukuoka, Japan; Department of Pathology, Nihon University School of Medicine, Tokyo, Japan; Department of Medicine and Clinical Science, Graduate School of Medical Science, Kyushu University, Fukuoka, Japan; Division of Nephrology, Department of Internal Medicine, University of Nagoya, Nagoya, Japan

**Keywords:** IgA nephropathy, Randomized controlled trial, Tonsillectomy, Steroid pulse therapy, Pathology

## Abstract

**Background:**

The IgA nephropathy (IgAN) Study Group in Japan conducted a multicenter, randomized, controlled trial of tonsillectomy with steroid pulse therapy (TSP) versus steroid pulse monotherapy in patients with IgAN (UMIN Clinical Trial Registry Number; C000000384). The effects of therapies in relation to pathological severity were analyzed in this study.

**Methods:**

The patients with IgAN, urinary protein 1.0–3.5 g/day, serum creatinine of 1.5 mg/dl or less were randomly assigned to receiving TSP (Group A) or steroid pulses alone (Group B). The primary endpoint was the disappearance of proteinuria and/or hematuria. Twenty-six biopsies in Group A and 33 in Group B were available. The histological grades (HG) according to the percentage of glomeruli with crescent or sclerosis and the Oxford classification were analyzed.

**Results:**

The patients in Group A had a 4.32- to 12.1-fold greater benefit of disappearance of proteinuria and 3.61- to 8.17-fold greater benefit of clinical remission (disappearance of proteinuria and hematuria) than those in Group B in patients with HG2–3, acute lesions (cellular or fibrocellular crescent) affecting more than 5 % of glomeruli, chronic lesions (fibrous crescents or sclerosis) affecting more than 20 % and S1. In contrast, odds ratios for disappearance of proteinuria or clinical remission in Group A to Group B were not significant in patients with HG 1, acute lesion in 5 % or less of glomeruli, chronic lesion in 20 % or less and S0. The disappearance of hematuria showed no relation to pathological severity.

**Conclusion:**

TSP might be better employed according to the pathological severity.

**Electronic supplementary material:**

The online version of this article (doi:10.1007/s10157-015-1159-2) contains supplementary material, which is available to authorized users.

## Introduction

In 2002, Hotta et al. [1] reported the significant effect of tonsillectomy and steroid pulse therapy on the remission of urinary abnormalities in IgA nephropathy (IgAN). Since then, tonsillectomy with steroid pulse therapy (TSP) has become one of the most widely used therapy protocols in adult patients with IgAN, and is being performed in ~40 and 70 % of the institutions in Japan according to a survey in 2006 [2] and 2008 [3], respectively. Imai et al. [4] mentioned in their review article that Japanese nephrologists face a treatment dilemma in adult IgAN such as the risk of overtreatment with TSP versus the loss of a “golden period” of the effect of TSP due to late intervention. Internationally, TSP has not yet been accepted as the standard therapy in patients with IgAN because of a lack of randomized control trial (RCT) [5, 6].

Recently, the Special IgAN Study Group of the Progressive Glomerular Diseases Study Committee organized by the Ministry of Health, Labor and Welfare of Japan conducted a multicenter RCT of TSP versus steroid pulse monotherapy in patients with IgAN. They reported that anti-proteinuric effect was significantly higher in patients receiving TSP than in those treated with steroid pulses alone, although the difference was marginal [7]. The study protocol did not include detail pathological analyses. Here, we reported the results of pathological sub-analysis of the RCT. The aim of this study was to examine the differences in the effect on the remission of urinary abnormality between TSP and steroid pulse monotherapy in relation to pathological severity.

## Subjects and methods

### Patients and study protocol

The inclusion criteria of the original RCT were biopsy-proven IgAN, age ranged from 10 to 69 years, urinary protein excretion ranging 1.0–3.5 g/day, serum creatinine of 1.5 mg/dl or less. Exclusion criteria were nephrotic syndrome, serum creatinine of more than 1.5 mg/dl, recent treatment with corticosteroids and/or immunosuppressive agents, and contraindications for general anesthesia and/or tonsillectomy as assessed by otolaryngologists. Steroid protocol was three courses of 500 mg of methylprednisolone for 3 consecutive days every 2 months. Oral prednisolone (0.5 mg/Kg) was given every other day for 6 months. The entire trial period (treatment + follow-up) was 12 months. The participants were randomly assigned to receiving TSP (Group A) or steroid pulses alone (Group B). In Group A, one to 3 weeks after tonsillectomy, the steroid protocol was employed.

Among 72 patients of original RCT, the patients who had been biopsied within 1 year before the entry to RCT and those in whom we could obtain informed consent were included in this pathological sub-analysis. IRB/Ethics Committee approval of Jikei University school of Medicine has been obtained. The IRB approval number was 23–044 (6505). Twelve patients had been biopsied more than 1 year before the entry to RCT. In one patient, we could not get informed consent. Finally, 59 patients were included in this study. Twenty-six biopsies in Group A and 33 in Group B were analyzed.

Clinical parameters included blood pressure, urinary protein excretion, serum creatinine, and urinary sediment with red blood cells. Estimated glomerular filtration rate (eGFR) was computed using the following equation [8]: eGFR (ml/min/1.73 m^2^) = 194 × (serum creatinine in mg/dl)^−1.094^ × (age in years)^−0.287^ (×0.739 if female).

The primary endpoints were the disappearance of proteinuria and/or hematuria 12 months after the initial treatment. The disappearance of proteinuria was defined as urinary protein excretion of <0.3 g in a 24-h urine collection or <0.3 g/g creatinine in urine samples at visits. The disappearance of hematuria was defined as a number of red blood cells in urinary sediments of <5 per high power field. Clinical remission was defined as the disappearance of both proteinuria and hematuria.

### Pathological parameters

We analyzed histological grades (HG) of the 3rd version clinical guideline for IgAN in Japan [9]. HG1-4 was established corresponding to <25 %, 25–49 %, 50–74 % and ≥75 % of glomeruli exhibiting crescents, segmental sclerosis or global sclerosis [9, 10]. Cellular or fibrocellular crescents were defined as acute lesions and fibrous crescents, segmental or global sclerosis were defined as chronic lesions. Activity was described in each biopsy as follows: only acute lesions as A, both acute and chronic lesions as A/C, and only chronic lesions as C. Furthermore, according to the percentage of glomeruli with the acute lesions were categorized as 5 % or less and more than 5 %. Chronic lesions were also categorized as 20 % or less and more than 20 % of the glomeruli. We also evaluated the Oxford classification [11]. All pathological lesions were defined according to the definition of Oxford classification [12]. Five pathologists (RK, KJ, AH, SH, AS) rated all the biopsies. HG and activity were determined according to the consensus of five pathologists.

### Statistical analysis

SPSS ver. 21 was used for the analysis. For comparing the parameters between the two groups, the unpaired *t* test and Wilcoxon rank-sum test were used for normally and non-normally distributed variables, respectively. The difference in frequency was evaluated using Pearson’s Chi-square test. The impacts of each treatment on the disappearance of proteinuria and/or hematuria were analyzed by logistic regression analysis according to the severity of HG, category of acute and chronic lesions, and the Oxford classification. The heterogeneity of the effect of treatment groups on the disappearance of proteinuria and/or hematuria between the categories of each pathological parameter was analyzed by the test for interaction. Results were presented as odds ratios (ORs) with 95 % confidence intervals (CIs) and *P* values. *P* < 0.05 was considered statistically significance in all analyses.

## Results

### Baseline clinical characteristics

Baseline clinical characteristics are summarized in Table 1. There were no significant differences in age, gender distribution, blood pressure, the percentage of patients given renin–angiotensin system (RAS) inhibitors, urinary protein excretion, urinary red blood cell score, and eGFR between the two groups.

**Table 1:** Baseline clinical characteristics

Parameter	Group A	Group B	*p*
	Tonsillectomy + steroid pulses	Steroid pulses alone	
Number of patients	26	33	
Age (y.o.)	37 ± 14	41 ± 14	n.s.
Period from onset to start of treatment (years)	4.6 ± 5.4	5.0 ± 5.5	n.s.
Men (%)	12 (46)	15 (46)	n.s.
SBP (mmHg)	117 ± 13	121 ± 10	n.s.
DBP (mmHg)	69 ± 10	73 ± 8	n.s.
MBP (mmHg)	85 ± 10	89 ± 8	n.s.
Patients with RASi (%)	12 (46)	16 (49)	n.s.
UP-UCRE (g/gcr)	1.7 ± 1.1	1.7 ± 1.0	n.s.
URBC score (median, range)	3 (1–5)	2 (1–5)	n.s.
eGFR	74 ± 26	67 ± 22	n.s.

URBC score; urinary RBC 0–1/HPF = 0, 1–4/HPF = 1, 5–19/HPF = 2, 20–49/HPF = 3, 50–99/HPF = 4, >100 = 5

*SBP* systolic blood pressure, *DBP* diastolic blood pressure, *MBP* mean blood pressure, *RASi* renin–angiotensin system inhibitor, *UP-UCRE* urinary protein–creatinine ratio, *URBC* urinary red blood cell, *eGFR* estimated glomerular filtration rate

### Pathological parameters

Pathological characteristics are summarized in Table 2. There were no statistical significant differences in HG, activity, the percentage of glomeruli with acute and chronic lesions, and Oxford classification between the two groups.

**Table 2:** Pathological characteristics

Parameter	Category	Group A		Group B		*p*
		Tonsillectomy + steroid pulses		Steroid pulses alone		
		*n*	%	*n*	%	
Histological Grade	HG 1	13	50	17	52	n.s.
	HG 2	8	31	14	42	
	HG 3	5	19	2	6	
	HG 4	0	0	0	0	
Activity	Null	3	12	2	6	n.s.
	A	0	0	0	0	
	A/C	17	65	25	76	
	C	6	23	6	18	
Acute lesion %, median (range)		4.8 (0–16.7)		7.3 (0–35.3)		n.s.
Chronic lesion %, median (range)		29.4 (0–72.4)		27.3 (0–63.6)		n.s.
Oxford classification						
M	1	10	39	15	46	n.s.
E	1	12	46	14	42	n.s.
S	1	21	81	30	91	n.s.
T	1–2	6	23	10	30	n.s.

*HG* histological grade, *A* only acute lesions, *A/C* acute and chronic lesions, *C* only chronic lesions, *M1* mesangial hypercellularity score more than 0.5, *E1* presence of endocapillary hypercellularity, *S1* presence of segmental glomerulosclerosis, *T1*–*2* Tubular atrophy/interstitial fibrosis involving cortical area more than 25 %

### Impact of steroid pulses and tonsillectomy on proteinuria

The percentage of the disappearance of proteinuria in each pathological category in each treatment group and the impacts of each treatment on the disappearance of proteinuria 12 months after initiation of treatment according to the statuses of each pathological parameter are shown in Table 3.

**Table 3:** Odds ratio for the disappearance of proteinuria in Group A versus Group B according to the statuses of each pathological parameter

Subgroup	Intention to treated analysis					
	N (% of disappearance of proteinuria)					
	Group A	Group B	OR (A vs B)	95 % CI	*p*	*p* for heterogeneity
	Tonsillectomy + steroid pulses	Steroid pulses alone				
Histological grade						
HG 1	13 (54 %)	17 (53 %)	1.04	0.24–4.41	0.961	0.093
HG 2–3	13 (69 %)	16 (25 %)	6.75	1.32–34.6	0.022	
Acute lesion						
≤5 %	14 (36 %)	12 (25 %)	1.67	0.30–9.16	0.557	0.165
>5 %	12 (92 %)	21 (48 %)	12.1	1.32–111	0.028	
Chronic lesion						
≤20 %	11 (54 %)	12 (67 %)	0.60	0.11–3.23	0.553	0.020
>20 %	15 (67 %)	21 (24 %)	6.40	1.47–27.8	0.013	
Oxford classification						
Mesangial hypercellularity						
M0	16 (56 %)	18 (44 %)	1.61	0.41–6.24	0.493	0.341
M1	10 (70 %)	15 (33 %)	4.67	0.83–26.2	0.080	
Endocapillary proliferation						
E0	14 (50 %)	19 (26 %)	2.80	0.65–12.1	0.168	0.848
E1	12 (75 %)	14 (57 %)	2.25	0.42–12.1	0.345	
Segmental sclerosis						
S0	5 (20 %)	3 (67 %)	0.13	0.01–3.23	0.210	0.045
S1	21 (71 %)	30 (37 %)	4.32	1.30–14.4	0.017	
Tubular atrophy/Interstitial fibrosis						
T0	20 (55 %)	23 (48 %)	1.33	0.40–4.44	0.639	0.068
T1–2	6 (83 %)	10 (20 %)	20.0	1.42–282	0.027	

*N* number of patients, *HG* histological grade, *OR* odds ratio, *CI* confidence interval, *M0* mesangial hypercellularity score 0.5 or less, *M1* mesangial hypercellularity score more than 0.5, *E0* absence of endocapillary hypercellularity, *E1* presence of endocapillary hypercellularity, *S0* absence of segmental glomerulosclerosis, *S1* presence of segmental glomerulosclerosis, *T0* Tubular atrophy/interstitial fibrosis involving cortical area 25 % or less, *T1–2* Tubular atrophy/interstitial fibrosis involving cortical area more than 25 %

In the patients classified in HG 1, OR for the disappearance of proteinuria in Group A compared to Group B was not significant. In contrast, in HG 2–3, the patients in Group A had a 6.75-fold (95 % CI: 1.32–34.6) greater benefit of the disappearance of proteinuria than those in Group B. The effect of treatment in both groups on the disappearance of proteinuria tended to be heterogeneous between HG 1 and HG 2–3 (*p* for heterogeneity = 0.093). Similarly, although OR was not significant in the patients with acute lesions 5 % or less of glomeruli, Group A had a 12.1-fold (95 % CI: 1.32–111) greater benefit of the disappearance of proteinuria than Group B in the patients with acute lesions more than 5 % of glomeruli. In the patients with chronic lesions more than 20 % of glomeruli, Group A had a 6.4-fold (95 % CI: 1.47–27.8) greater benefit of the disappearance of proteinuria than Group B, whereas the OR of Group A to Group B was not significant in the patients with chronic lesions 20 % or less of glomeruli. Moreover, there was a significant heterogeneity of the effect of both treatments between the categories of chronic lesions (*p* for heterogeneity = 0.02).

As for Oxford classification, the ORs of Group A to Group B were not significant in patients with both M0 and M1, and those with both E0 and E1. In patients with S1, Group A had a 4.32-fold (95 % CI: 1.30–14.4) greater benefit of the disappearance of proteinuria than Group B, whereas the OR of Group A to Group B was not significant in patients with S0. There was a significant heterogeneity of the effect of both treatments between S0 and S1 (*p* for heterogeneity = 0.045). Similarly, Group A had a 20.0-fold (95 % CI: 1.42–282) greater benefit of the disappearance of proteinuria than Group B in patients with T1–2, whereas the OR of Group A to Group B was not significant in patients with T0. The effect of treatments tended to be heterogeneous between T0 and T1–2 (*p* for heterogeneity = 0.068).

### Impact of steroid pulses and tonsillectomy on hematuria

The percentage of the disappearance of hematuria in each pathological category in each treatment group and the impacts of each treatment on the disappearance of hematuria 12 months after initiation of treatment according to the statuses of each pathological parameter are shown in Table 4. ORs for the disappearance of hematuria in Group A compared to Group B were not significant in any category of any pathological parameters.

**Table 4:** Odds ratio for the disappearance of hematuria in Group A versus Group B according to the statuses of each pathological parameter

Subgroup	Intention to treated analysis					
	N (% of disappearance of hematuria)					
	Group A	Group B	OR (A vs B)	95 % CI	*p*	*p* for heterogeneity
	Tonsillectomy + steroid pulses	Steroid pulses alone				
Histological grade						
HG 1	13 (62 %)	17 (71 %)	0.67	0.15–3.08	0.603	0.157
HG 2–3	13 (77 %)	16 (50 %)	3.33	0.66–16.8	0.145	
Acute lesion						
≤5 %	14 (64 %)	12 (58 %)	1.29	0.26–6.27	0.756	0.751
>5 %	12 (75 %)	21 (62 %)	1.85	0.38–8.93	0.446	
Chronic lesion						
≤20 %	11 (55 %)	12 (67 %)	0.60	0.11–3.25	0.553	0.166
>20 %	15 (80 %)	21 (57 %)	3.00	0.65–13.9	0.160	
Oxford classification						
Mesangial hypercellularity						
M0	16 (81 %)	18 (78 %)	1.24	0.23–6.62	0.803	0.872
M1	10 (50 %)	15 (40 %)	1.50	0.30–7.53	0.622	
Endocapillary proliferation						
E0	14 (71 %)	19 (63 %)	1.46	0.33–6.46	0.619	0.980
E1	12 (67 %)	14 (57 %)	1.50	0.30–7.43	0.619	
Segmental sclerosis						
S0	5 (60 %)	3 (67 %)	0.75	0.04–15.0	0.851	0.851
S1	21 (71 %)	30 (60 %)	1.67	0.50–5.51	0.402	
Tubular atrophy/Interstitial fibrosis						
T0	20 (70 %)	23 (61 %)	1.50	0.42–5.35	0.532	0.926
T1–2	6 (67 %)	10 (60 %)	1.33	0.16–11.1	0.790	

*N* number of patients, *HG* histological grade, *OR* odds ratio, *CI* confidence interval, *M0* mesangial hypercellularity score 0.5 or less, *M1* mesangial hypercellularity score more than 0.5, *E0* absence of endocapillary hypercellularity, *E1* presence of endocapillary hypercellularity, *S0* absence of segmental glomerulosclerosis, *S1* presence of segmental glomerulosclerosis, *T0* tubular atrophy/interstitial fibrosis involving cortical area 25 % or less, *T1*–*2* tubular atrophy/interstitial fibrosis involving cortical area more than 25 %

### Impact of steroid pulses and tonsillectomy on the clinical remission

The percentage of the clinical remission in each pathological category in each treatment group and the impacts of each treatment on the clinical remission 12 months after initiation of treatment according to the statuses of each pathological parameter are shown in Table 5. In the patients classified in HG 1, OR for the clinical remission in Group A compared to Group B was not significant. In contrast, in HG 2–3, the patients in Group A had a 8.17-fold (95 % CI: 1.30–51.4) greater benefit of the clinical remission than those in Group B. The effect of both treatments tended to be heterogeneous between HG 1 and HG 2–3 (*p* for heterogeneity = 0.066). Similarly, although OR was not significant in the patients with acute lesions 5 % or less of glomeruli, Group A had a 5.00-fold (95 % CI: 1.08–23.1) greater benefit of the clinical remission than Group B in the patients with acute lesions more than 5 % of glomeruli. In the patients with chronic lesions more than 20 % of glomeruli, Group A had a 6.86-fold (95 % CI: 1.40–33.6) greater benefit of the clinical remission than Group B, whereas the OR of Group A to Group B was not significant in the patients with chronic lesions 20 % or less of glomeruli. Moreover, there was a significant heterogeneity of the effect of both treatments on the clinical remission between the categories of chronic lesions (*p* for heterogeneity = 0.035).

**Table 5:** Odds ratio for the clinical remission in Group A versus Group B according to the statuses of each pathological parameter

Subgroup	Intention to treated analysis							
	*N* (% of clinical remission)							
	Group A	Group B	OR (A vs B)		95 % CI		*p*	*p* for heterogeneity
	Tonsillectomy + steroid pulses	Steroid pulses alone						
Histological grade								
HG 1	13 (39 %)	17 (41 %)	0.89		0.20–3.91		0.880	0.066
HG 2–3	13 (54 %)	16 (13 %)	8.17		1.30–51.4		0.025	
Acute lesion								
≤5 %	14 (29 %)	12 (25 %)	1.20		0.21–6.88		0.838	0.228
>5 %	12 (67 %)	21 (29 %)	5.00		1.08–23.1		0.039	
Chronic lesion								
≤20 %	11 (36 %)	12 (50 %)	0.57		0.11–3.04		0.511	0.035
>20 %	15 (53 %)	21 (14 %)	6.86		1.40–33.6		0.018	
Oxford classification								
Mesangial hypercellularity								
M0	16 (44 %)	18 (39 %)	1.22		0.31–4.80		0.774	0.167
M1	10 (50 %)	15 (13 %)	6.50		0.94–45.1		0.058	
Endocapillary proliferation								
E0	14 (36 %)	19 (16 %)	2.96		0.57–15.4		0.196	0.690
E1	12 (58 %)	14 (43 %)	1.87		0.39–8.89		0.433	
Segmental sclerosis								
S0	5 (20 %)	3 (67 %)	0.13		0.01–3.23		0.210	0.057
S1	21 (52 %)	30 (23 %)	3.61		1.08–12.0		0.036	
Tubular atrophy/interstitial fibrosis								
T0	20 (45 %)	23 (35 %)	1.53		0.45–5.25		0.495	0.230
T1–2	6 (50 %)	10 (10 %)	9.00		0.66–123		0.099	

*N* number of patients, *HG* histological grade, *OR* odds ratio, *CI* confidence interval, *M0* mesangial hypercellularity score 0.5 or less, *M1* mesangial hypercellularity score more than 0.5, *E0* absence of endocapillary hypercellularity, *E1* presence of endocapillary hypercellularity, *S0* absence of segmental glomerulosclerosis, *S1* presence of segmental glomerulosclerosis, *T0* Tubular atrophy/interstitial fibrosis involving cortical area 25 % or less, *T1*–*2* Tubular atrophy/interstitial fibrosis involving cortical area more than 25 %

As for Oxford classification, the ORs of Group A to Group B were not significant in patients with both M0 and M1, and those with both E0 and E1. In patients with S1, Group A had a 3.61-fold (95 % CI: 1.08–12.0) greater benefit of the clinical remission than Group B, whereas the OR of Group A to Group B was not significant in patients with S0. The effect of both treatments tended to be heterogeneous between S0 and S1 (*p* for heterogeneity = 0.057). The ORs of Group A to Group B were not significant in patients with both T0 and T1–2.

### Per protocol-based analysis

One patient who had assigned to Group A did not undergo tonsillectomy, and two patients who had assigned to Group B underwent tonsillectomy. Thus, 27 patients received TSP and 32 received steroid pulse monotherapy. We also performed per protocol-based (PPB) analyses and the results are almost similar to those of intention to treat analysis except for the following results. The heterogeneity of the effect of both treatment on the disappearance of proteinuria and on the clinical remission was significant between HG 1 and HG 2–3 in the PPB analysis (*p* for heterogeneity = 0.043 and 0.012, respectively). Similarly, the heterogeneity of the effect of both treatments on the clinical remission between S0 and S1 was significant in the PPB analysis (p for heterogeneity = 0.042). The results of PPB analyses are shown in supplemental Tables 1 to 5.

## Discussion

The original RCT of this pathological sub-analysis is the 1st one in the world [7]. They found that the anti-proteinuric effect was significantly greater in TSP than in the steroid pulse alone, even though the difference was marginal. The patients of this RCT had urinary protein excretion ranging from 1.0 to 3.5 g/day, which is the definite risk factor of progression to end-stage renal failure (ESRF) in IgAN [13, 14]. Thus, the majority of the patients included in this study are at a high risk to progress to ESRF. Therefore, the results of this study should not be applied to the patients with mild IgAN.

In the present pathological sub-analysis, we found that the effect of both treatments on the disappearance of proteinuria and on the clinical remission was heterogeneous according to the pathological severity. Namely, the superior effect of Group A compared to Group B on the disappearance of proteinuria and on the clinical remission was evident in patients with HG 2–3, acute lesions in more than 5 % of glomeruli, chronic lesions in more than 20 % of glomeruli, and S1. On the other hand, the effect of treatments on the disappearance of proteinuria and on the clinical remission in Group A and Group B was comparable in the patients with HG 1, acute lesions in 5 % or less of glomeruli, chronic lesions in 20 % or less of glomeruli and S0. These results suggest that indication of TSP should be determined according to the pathological severity. TSP could be a valuable option for treatment in high-risk patients with severe pathological changes. Chronic lesions in more than 20 % of glomeruli may be the most reliable indicator for TSP because the effect of both treatments on the disappearance of proteinuria and on the clinical remission showed a significant heterogeneity between the categories of chronic lesions (*p* = 0.02, *p* = 0.035, respectively). It is not likely that TSP has a direct effect on chronic lesions themselves because chronic lesions may be an accumulation of irreversible scars from acute lesions. Therefore, the patients with extensive chronic lesions are highly likely to have had a long history of repeated exacerbation of acute inflammation. The hallmark of IgAN is underglycosylation in the hinge region of IgA1 and increasing evidence supports the underglycosylated IgA-containing immune-complex including IgG antibodies against the glycans of the hinge region of IgA1 are key factors for mesangial deposition and then trigger inflammation and glomerular injury [15]. Aberrantly glycosylated IgA1 may be synthesized in response to a mucosal infection including tonsil and thus abnormalities in the mucosal response to common microbial or food antigen may be involved in production of galactose-deficient IgA1 [16]. TSP might be effective probably due to the removal of potential antigenic stimuli by tonsillectomy and suppression of the abnormal immune response and subsequent acute inflammation by steroid pulse therapy. Kawasaki et al. [17] reported the effectiveness of TSP on remission of urinary abnormality in 11 pediatric patients who had been resistant to steroid therapy and suggested that TSP may be effective as rescue treatment for steroid-resistant IgAN in childhood. HG also may be a valuable marker for the indication of TSP because the effect of both treatments on the disappearance of proteinuria and on the clinical remission tended to be heterogeneous between HG 1 and HG 2–3. In addition, this heterogeneity was significant in PPB analysis. HG of the 3rd version clinical guideline for IgAN proposed by the Special IgAN Study Group in Japan is an evidence-based histological classification which can identify the magnitude of the risk of progression to ESRF [9, 10]. Since HG is a lumped system classification according to the percentage of glomeruli with both acute and chronic lesions, it might be the useful pathological parameter to determine the indication of TSP. HG 2 and HG 3 might be another good indication for TSP. As for the Oxford classification, only S lesion showed a significant heterogeneity between S0 and S1 of the effect of both treatments on the disappearance of proteinuria (*p* = 0.045). Since segmental sclerosis is included in chronic lesions and S0 and S1 is merely the absence or presence of segmental sclerosis, Oxford classification might not be as useful as the percentage of chronic lesions or HG to determine the indication of TSP.

In the patients with less severe pathological changes, TSP might not necessarily be applied. Such patients might be amenable to treatment with steroid pulse alone. The high level evidence of the effect of steroid pulse therapy to prevent the progression of IgAN has been reported by Pozzi et al. [18, 19]. Their RCT and its follow-up study showed that the risk of a doubling in serum creatinine after 10 years was found to be significantly lower in the patients receiving steroid pulses than those receiving supportive therapy alone. We have previously reported that steroid pulse therapy rather than oral steroid was significantly effective on preventing the progression to ESRF in a retrospective cohort study of 702 patients with IgAN [20].

The concept that TSP should not necessarily be indicated to all the patients with IgAN also mentioned in the report by Miyazaki et al. [21]. They reported an effect on clinical remission of TSP compared to steroid monotherapy in multicenter prospective cohort study in 101 patients with IgAN observed for 5 years and concluded that the goal should be cure and release from disease at an earlier stage of IgAN. In their study, the difference of efficacy on clinical remission between TSP and steroid monotherapy was significant only in the patients with proteinuria 1.0 g/day or more. They suggested that it is necessary to reveal which clinical or histological features will most likely derive the most benefit from TSP and the best strategy for each stage of IgAN should be confirmed.

The comparison of the effect on the remission of proteinuria between TSP and steroid pulse monotherapy in relation to pathological severity has been reported in only one study by Komatsu et al. [22]. They employed the histological grading of the 2nd version clinical guideline for IgAN in Japan [23] as follows: Grade 1; mild mesangial proliferation, no crescent and no sclerosis, Grade 2; mild mesangial proliferation, crescent or sclerosis less than 10 % of glomeruli, Grade 3; moderate mesangial proliferation, crescent or sclerosis from 10 to 30 % of glomeruli, and Grade 4; severe mesangial proliferation, crescent or sclerosis more than 30 % of glomeruli. The therapeutic effect on the remission of proteinuria was almost equivalent between TSP and steroid pulse monotherapy in Grade 2, which is compatible with HG 1. In contrast, the patients in TSP group showed a significantly higher remission of proteinuria than those in steroid pulse monotherapy in Grade 3, which is compatible HG 1 and part of HG 2. These results are interestingly similar to our results. On the other hand, no patients attained remission of proteinuria in both TSP and steroid pulse alone in the patients with Grade 4, which is compatible with HG 2 or more. This finding is not compatible with our results. We found superior effect of TSP to steroid pulse monotherapy in HG 2–3. The reason for the disparity in the results might be due to a difference in the treatment protocol: steroid pulse therapy was administered as one course in their study and as three courses in our study.

Very recently, Miyamoto et al. reported that TSP had an impact on achieving clinical remission in only the group with histological grade 3 of the 2nd version clinical guideline for IgAN in Japan and M1 in their multicenter retrospective cohort study in 284 patients with IgAN who received TSP or corticosteroid therapy or conservative therapy [24]. In their study, the effect of TSP was compared to that of conservative therapy and they also found a significant superior effect of corticosteroid therapy including steroid pulses compared to that of conservative therapy in the group with histological grade 3. Although the study design and the compared treatment groups are different to our study, it is of interest that they found the difference of superior effect of TSP to conservative therapy according to the histological severity.

In contrast to the heterogeneous effect of the two treatments on disappearance of proteinuria and on clinical remission between the categories of several pathological parameters, the effects on the disappearance of hematuria were comparable in both treatment groups irrespective of the severity of any pathological parameter. Thus, the therapeutic advantage of TSP in terms of disappearance of hematuria was not observed at least in short-term observation.

The limitations of the present pathological sub-analyses are as follows. First, since this study is the post hoc analysis of the RCT and not all biopsies in the original RCT were available, the results of this study might not fully reflect the original RCT. Second, in this analysis only the short-term clinical response on urinary abnormality was analyzed. The analyses of long-term outcome are required to confirm the effect of TSP. An analysis of long-term follow-up data in the original RCT is in progress. Third, since there were no patients with HG 4 in the present study, the superior effect of TSP to steroid pulse therapy on proteinuria in the patients with very severe histological changes, such as crescent or sclerosis in more than 75 % of glomeruli, remains to be elucidated.

In conclusion, TSP may have a therapeutic advantage compared to steroid pulse monotherapy in the patients with Histological Grade 2 and 3, acute lesions affecting more than 5 % of glomeruli, and chronic lesions affecting more than 20 % of glomeruli, but not in those with less severe lesions. Thus, TSP might be better employed according to the pathological severity.

## Electronic supplementary material

Below is the link to the electronic supplementary material.

Supplementary material 1 (DOCX 15 kb)

Supplementary material 2 (DOCX 17 kb)

Supplementary material 3 (DOCX 22 kb)

Supplementary material 4 (DOCX 21 kb)

Supplementary material 5 (DOCX 21 kb)
