# Supplementary material for: Pathological sub-analysis of a multicenter randomized controlled trial of tonsillectomy combined with steroid pulse therapy versus steroid pulse monotherapy in patients with immunoglobulin A nephropathy
Source: Clin Exp Nephrol. 2015 Sep 9;20:244–52. doi: 10.1007/s10157-015-1159-2 (PMC4819588; doi:10.1007/s10157-015-1159-2)
Supplement: Supplementary file 1 — Supplementary material 1 (DOCX 15 kb) [file 10157_2015_1159_MOESM1_ESM.docx]

| Supplemental Table 1. Baseline clinical characteristics | | | | | | | | | |
| --- | --- | --- | --- | --- | --- | --- | --- | --- | --- |
| Parameter | Group A | | |  | Group B | | |  | p |
|  | Tonsillectomy+ steroid pulses | | |  | Steroid pulses alone | | |  |  |
| number of patients | 27 | | |  | 32 | | |  | n.s. |
| Age (y.o.) | 37 | ± | 14 |  | 41 | ± | 14 |  | n.s. |
| Period from onset to start of treatment (years) | 4.9 | ± | 5.3 |  | 4.8 | ± | 5.6 |  | n.s. |
| Men (%) | 11 | (41) | |  | 16 | (50) | |  | n.s. |
| SBP (mmHg) | 118 | ± | 12 |  | 120 | ± | 11 |  | n.s. |
| DBP (mmHg) | 70 | ± | 10 |  | 72 | ± | 9 |  | n.s. |
| MBP (mmHg) | 86 | ± | 10 |  | 88 | ± | 8 |  | n.s. |
| Patients with RASi (%) | 12 | (44) | |  | 16 | (50) | |  | n.s. |
| UP-UCRE (g/gcr) | 1.5 | ± | 0.5 |  | 1.6 | ± | 0.6 |  | n.s. |
| URBC score (median,range) | 3 | (1-5) | |  | 2 | (1-5) | |  | n.s. |
| eGFR | 74 | ± | 25 |  | 67 | ± | 22 |  | n.s. |
|  |  |  |  |  |  |  |  |  |  |
| Abbreviations are: SBP; systolic blood pressure, DBP; diastolic blood pressure, MBP; mean blood pressure, RASi; renin-angiotensin system inhibitor, UP-UCRE; urinary protein creatinine ratio, URBC; urinary red blood cell, eGFR; estimated glomerular filtration rate. | | | | | | | | | |
| URBC score; urinary RBC 0-1/HPF=0, 1-4/HPF=1, 5-19/HPF=2, 20-49/HPF=3, 50-99/HPF=4, >100=5 | | | | | | | | | |
