# Supplementary material for: Pathological sub-analysis of a multicenter randomized controlled trial of tonsillectomy combined with steroid pulse therapy versus steroid pulse monotherapy in patients with immunoglobulin A nephropathy
Source: Clin Exp Nephrol. 2015 Sep 9;20:244–52. doi: 10.1007/s10157-015-1159-2 (PMC4819588; doi:10.1007/s10157-015-1159-2)
Supplement: Supplementary file 2 — Supplementary material 2 (DOCX 17 kb) [file 10157_2015_1159_MOESM2_ESM.docx]

| Supplemental Table 2. Pathological characteristics | | | | | | | | | | | | | |
| --- | --- | --- | --- | --- | --- | --- | --- | --- | --- | --- | --- | --- | --- |
|  |  |  |  |  |  |  |  |  |  |  |  |  |  |
| Parameter | Category |  | Group A | | | |  | Group B | | | |  | p |
|  |  |  | Tonsillectomy+ steroid pulses | | | |  | Steroid pulses alone | | | |  |  |
|  |  |  | n |  | % |  |  | n |  | % |  |  |  |
| Histological Grade | HG 1 |  | 14 | ( | 52 | ) |  | 16 | ( | 50 | ) |  | n.s. |
|  | HG 2 |  | 8 | ( | 30 | ) |  | 14 | ( | 44 | ) |  |  |
|  | HG 3 |  | 5 | ( | 19 | ) |  | 2 | ( | 6 | ) |  |  |
|  | HG 4 |  | 0 | ( | 0 | ) |  | 0 | ( | 0 | ) |  |  |
| Activity | Null |  | 3 | ( | 11 | ) |  | 2 | ( | 6 | ) |  | n.s. |
|  | A |  | 0 | ( | 0 | ) |  | 0 | ( | 0 | ) |  |  |
|  | A/C |  | 18 | ( | 67 | ) |  | 24 | ( | 75 | ) |  |  |
|  | C |  | 6 | ( | 22 | ) |  | 6 | ( | 19 | ) |  |  |
| Acute lesion%, median (range) | |  | 5.1 ( 0 - 16.7 ) | | | |  | 6.7 ( 0 - 35.3 ) | | | |  | n.s. |
| Chronic lesion%, median (range) | |  | 25.4 ( 0 - 72.4 ) | | | |  | 27.5 ( 0 - 63.6 ) | | | |  | n.s. |
| Oxford classification | |  |  |  |  |  |  |  |  |  |  |  |  |
| M | 1 |  | 12 | ( | 44 | ) |  | 13 | ( | 41 | ) |  | n.s. |
| E | 1 |  | 14 | ( | 52 | ) |  | 12 | ( | 38 | ) |  | n.s. |
| S | 1 |  | 22 | ( | 82 | ) |  | 29 | ( | 91 | ) |  | n.s. |
| T | 1-2 |  | 6 | ( | 22 | ) |  | 10 | ( | 31 | ) |  | n.s. |

Abbreviations are: HG; histological grade, A; only acute lesions, A/C; acute and chronic lesions, C; only chronic lesions, M1; mesangial hypercellularity score more than 0.5, E1; presence of endocapillary hypercellularity, S1; presence of segmental glomerulosclerosis, T1-2; Tubular atrophy/interstitial fibrosis involving cortical area more than 25%.
