# Supplementary material for: Pathological sub-analysis of a multicenter randomized controlled trial of tonsillectomy combined with steroid pulse therapy versus steroid pulse monotherapy in patients with immunoglobulin A nephropathy
Source: Clin Exp Nephrol. 2015 Sep 9;20:244–52. doi: 10.1007/s10157-015-1159-2 (PMC4819588; doi:10.1007/s10157-015-1159-2)
Supplement: Supplementary file 3 — Supplementary material 3 (DOCX 22 kb) [file 10157_2015_1159_MOESM3_ESM.docx]

| Supplemental Table 3. Odds ratio for the disappearance of proteinuria in Group A versus Group B according to the statuses of each pathological parameters in per protocol based analyses | | | | | | | | |
| --- | --- | --- | --- | --- | --- | --- | --- | --- |
|  |  |  |  |  |  |  |  |  |
|  |  | As treated analysis | | | | | | |
| Subgroup | | N (% of disappearance of proteiunria) | |  |  | | | |
|  |  | Group A | Group B |  | OR (A vs B) | 95% CI | p | p for heterogeneity |
|  |  | Tonsillectomy+ steroid pulses | Steroid pulses alone |  |  |  |  |  |
| Histological grade | |  |  |  |  |  |  |  |
|  | HG 1 | 14 (57%) | 16 (50%) |  | 1.33 | 0.32-5.64 | 0.696 | 0.043 |
|  | HG 2-3 | 13 (77%) | 16 (19%) |  | 14.4 | 2.39-87.4 | 0.004 |  |
| Acute lesion | |  |  |  |  |  |  |  |
|  | ≤5% | 13 (38%) | 13 (38%) |  | 2.08 | 0.38-11.5 | 0.399 | 0.133 |
|  | >5% | 14 (93%) | 19 (42%) |  | 17.9 | 1.93-166 | 0.011 |  |
| Chronic lesion | |  |  |  |  |  |  |  |
|  | ≤20% | 12 (58%) | 11 (64%) |  | 0.80 | 0.15-4.30 | 0.795 | 0.023 |
|  | >20% | 15 (73%) | 21 (19%) |  | 11.7 | 2.41-56.7 | 0.002 |  |
| Oxford classification | | |  |  |  |  |  |  |
| Mesangial hypercellularity | | |  |  |  |  |  |  |
|  | M0 | 15 (60%) | 19 (42%) |  | 2.06 | 0.52-8.18 | 0.303 | 0.178 |
|  | M1 | 12 (75%) | 13 (23%) |  | 10.0 | 1.59-62.7 | 0.014 |  |
| Endocapillary proliferation | | |  |  |  |  |  |  |
|  | E0 | 13 (54%) | 20 (25%) |  | 3.50 | 0.79-15.5 | 0.099 | 0.968 |
|  | E1 | 14 (79%) | 12 (50%) |  | 3.67 | 0.67-20.2 | 0.135 |  |
| Segmental sclerosis | |  |  |  |  |  |  |  |
|  | S0 | 5 (20%) | 3 (67%) |  | 0.13 | 0.01-3.23 | 0.210 | 0.021 |
|  | S1 | 22 (77%) | 29 (31%) |  | 7.56 | 2.12-26.9 | 0.002 |  |
| Tubular atrophy/Interstitial fibrosis | | |  |  |  |  |  |  |
|  | T0 | 21 (62%) | 22 (41%) |  | 2.35 | 0.69-7.98 | 0.172 | 0.150 |
|  | T1-2 | 6 (83%) | 10 (20%) |  | 20.0 | 1.42-282 | 0.027 |  |

Abbreviations are: N; number of patients, HG; histological grade, OR; odds ratio, CI; confidence interval, M0; mesangial hypercellularity score 0.5 or less, M1; mesangial hypercellularity score more than 0.5, E0; absence of endocapillary hypercellularity, E1; presence of endocapillary hypercellularity, S0; absence of segmental glomerulosclerosis, S1; presence of segmental glomerulosclerosis, T0; Tubular atrophy/interstitial fibrosis involving cortical area 25% or less, T1-2; Tubular atrophy/interstitial fibrosis involving cortical area more than 25%.
