# Supplementary material for: Pathological sub-analysis of a multicenter randomized controlled trial of tonsillectomy combined with steroid pulse therapy versus steroid pulse monotherapy in patients with immunoglobulin A nephropathy
Source: Clin Exp Nephrol. 2015 Sep 9;20:244–52. doi: 10.1007/s10157-015-1159-2 (PMC4819588; doi:10.1007/s10157-015-1159-2)
Supplement: Supplementary file 4 — Supplementary material 4 (DOCX 21 kb) [file 10157_2015_1159_MOESM4_ESM.docx]

| Supplemental Table 4. Odds ratio for the disappearance of hematuria in Group A versus Group B according to the statuses of each pathological parameters in per protocol based analyses | | | | | | | | | |
| --- | --- | --- | --- | --- | --- | --- | --- | --- | --- |
|  |  | As treated analysis | | | | | | | |
| Subgroup | | N (% of disappearance of hematuria) | | |  |  | | | |
|  |  | Group A | | Group B |  | OR (A vs B) | 95% CI | p | p for heterogeneity |
|  |  | Tonsillectomy+ steroid pulses | | Steroid pulses alone |  |  |  |  |  |
| Histological grade | |  | |  |  |  |  |  |  |
|  | HG 1 | 14 (57%) | | 16 (75%) |  | 0.44 | 0.09-2.09 | 0.305 | 0.078 |
|  | HG 2-3 | 13 (77%) | | 16 (50%) |  | 3.33 | 0.66-16.8 | 0.145 |  |
| Acute lesion | |  | |  |  |  |  |  |  |
|  | ≤5% | 13 (62%) | | 13 (62%) |  | 1.00 | 0.21-4.86 | 1.000 | 0.733 |
|  | >5% | 14 (71%) | | 19 (63%) |  | 1.46 | 0.33-6.46 | 0.619 |  |
| Chronic lesion | |  | |  |  |  |  |  |  |
|  | ≤20% | 12 (50%) | | 11 (73%) |  | 0.36 | 0.07-2.15 | 0.270 | 0.079 |
|  | >20% | 15 (80%) | | 21 (57%) |  | 3.00 | 0.65-13.9 | 0.160 |  |
| Oxford classification | | | |  |  |  |  |  |  |
| Mesangial hypercellularity | | | |  |  |  |  |  |  |
|  | M0 | 15 (80%) | | 19 (79%) |  | 1.07 | 0.20-5.71 | 0.940 | 0.731 |
|  | M1 | 12 (50%) | | 13 (39%) |  | 1.60 | 0.33-7.85 | 0.562 |  |
| Endocapillary proliferation | | | |  |  |  |  |  |  |
|  | E0 | 13 (69%) | | 20 (65%) |  | 1.21 | 0.27-5.40 | 0.801 | 0.957 |
|  | E1 | 14 (64%) | | 12 (58%) |  | 1.29 | 0.26-6.27 | 0.756 |  |
| Segmental sclerosis | | |  |  |  |  |  |  |  |
|  | S0 | 5 (60%) | | 3 (67%) |  | 0.75 | 0.04-15.0 | 0.851 | 0.734 |
|  | S1 | 22 (68%) | | 29 (62%) |  | 1.31 | 0.41-4.22 | 0.651 |  |
| Tubular atrophy/Interstitial fibrosis | | | |  |  |  |  |  |  |
|  | T0 | 21 (67%) | | 22 (64%) |  | 1.14 | 0.33-4.01 | 0.835 | 0.902 |
|  | T1-2 | 6 (67%) | | 10 (60%) |  | 1.33 | 0.16-11.1 | 0.790 |  |

Abbreviations are: N; number of patients, HG; histological grade, OR; odds ratio, CI; confidence interval, M0; mesangial hypercellularity score 0.5 or less, M1; mesangial hypercellularity score more than 0.5, E0; absence of endocapillary hypercellularity, E1; presence of endocapillary hypercellularity, S0; absence of segmental glomerulosclerosis, S1; presence of segmental glomerulosclerosis, T0; Tubular atrophy/interstitial fibrosis involving cortical area 25% or less, T1-2; Tubular atrophy/interstitial fibrosis involving cortical area more than 25%.
