# Supplementary material for: Pathological sub-analysis of a multicenter randomized controlled trial of tonsillectomy combined with steroid pulse therapy versus steroid pulse monotherapy in patients with immunoglobulin A nephropathy
Source: Clin Exp Nephrol. 2015 Sep 9;20:244–52. doi: 10.1007/s10157-015-1159-2 (PMC4819588; doi:10.1007/s10157-015-1159-2)
Supplement: Supplementary file 5 — Supplementary material 5 (DOCX 21 kb) [file 10157_2015_1159_MOESM5_ESM.docx]

| Supplemental Table 5. Odds ratio for the clinical remission in Group A versus Group B according to the statuses of each pathological parameters in per protocol based analyses | | | | | | | | |
| --- | --- | --- | --- | --- | --- | --- | --- | --- |
|  |  | As treated analysis | | | | | | |
| Subgroup | | N (% of clinical remission) | |  |  | | | |
|  |  | Group A | Group B |  | OR (A vs B) | 95% CI | p | p for heterogeneity |
|  |  | Tonsillectomy+ steroid pulses | Steroid pulses alone |  |  |  |  |  |
| Histological grade | |  |  |  |  |  |  |  |
|  | HG 1 | 14 (36%) | 16 (44%) |  | 0.71 | 0.16-3.12 | 0.654 | 0.012 |
|  | HG 2-3 | 13 (62%) | 16 (6%) |  | 24.0 | 2.38-242 | 0.007 |  |
| Acute lesion | |  |  |  |  |  |  |  |
|  | ≤5% | 13 (31%) | 13 (23%) |  | 1.48 | 0.26-8.50 | 0.659 | 0.297 |
|  | >5% | 14 (64%) | 19 (26%) |  | 5.04 | 1.13-22.5 | 0.034 |  |
| Chronic lesion | |  |  |  |  |  |  |  |
|  | ≤20% | 12 (33%) | 11 (55%) |  | 0.42 | 0.08-2.25 | 0.309 | 0.005 |
|  | >20% | 15 (60%) | 21 (10%) |  | 14.3 | 2.39-85.0 | 0.004 |  |
| Oxford classification | |  |  |  |  |  |  |  |
| Mesangial hypercellularity | | |  |  |  |  |  |  |
|  | M0 | 15 (47%) | 19 (37%) |  | 1.50 | 0.38-5.95 | 0.564 | 0.132 |
|  | M1 | 12 (50%) | 13 (8%) |  | 12.0 | 1.16-124 | 0.037 |  |
| Endocapillary proliferation | | |  |  |  |  |  |  |
|  | E0 | 13 (39%) | 20 (15%) |  | 3.54 | 0.67-18.6 | 0.135 | 0.582 |
|  | E1 | 14 (57%) | 12 (42%) |  | 1.87 | 0.39-8.89 | 0.433 |  |
| Segmental sclerosis | |  |  |  |  |  |  |  |
|  | S0 | 5 (20%) | 3 (67%) |  | 0.13 | 0.01-3.23 | 0.210 | 0.042 |
|  | S1 | 22 (55%) | 29 (21%) |  | 4.60 | 1.35-15.7 | 0.015 |  |
| Tubular atrophy/Interstitial fibrosis | | |  |  |  |  |  |  |
|  | T0 | 21 (48%) | 22 (32%) |  | 1.95 | 0.56-6.73 | 0.292 | 0.300 |
|  | T1-2 | 6 (50%) | 10 (10%) |  | 9.00 | 0.66-123 | 0.099 |  |

Abbreviations are: N; number of patients, HG; histological grade, OR; odds ratio, CI; confidence interval, M0; mesangial hypercellularity score 0.5 or less, M1; mesangial hypercellularity score more than 0.5, E0; absence of endocapillary hypercellularity, E1; presence of endocapillary hypercellularity, S0; absence of segmental glomerulosclerosis, S1; presence of segmental glomerulosclerosis, T0; Tubular atrophy/interstitial fibrosis involving cortical area 25% or less, T1-2; Tubular atrophy/interstitial fibrosis involving cortical area more than 25%.
